# Identification of a Six-Gene SLC Family Signature With Prognostic Value in Patients With Lung Adenocarcinoma

**DOI:** 10.3389/fcell.2021.803198

**Published:** 2021-12-15

**Authors:** Jing Zhu, Yong Mou, Shenglan Ye, Hongling Hu, Rujuan Wang, Qing Yang, Yi Hu

**Affiliations:** Department of Respiratory and Critical Care Medicine, The Central Hospital of Wuhan, Tongji Medical College, Huazhong University of Science and Technology, Wuhan, China

**Keywords:** solute carrier, signature, prognosis, lung adenocarcinoma, immune microenvironment

## Abstract

Given the importance of solute carrier (SLC) proteins in maintaining cellular metabolic homeostasis and that their dysregulation contributes to cancer progression, here we constructed a robust SLC family signature for lung adenocarcinoma (LUAD) patient stratification. Transcriptomic profiles and relevant clinical information of LUAD patients were downloaded from the TCGA and GEO databases. SLC family genes differentially expressed between LUAD tissues and adjacent normal tissues were identified using *limma* in R. Of these, prognosis-related SLC family genes were further screened out and used to construct a novel SLC family-based signature in the training cohort. The accuracy of the prognostic signature was assessed in the testing cohort, the entire cohort, and the external GSE72094 cohort. Correlations between the prognostic signature and the tumor immune microenvironment and immune cell infiltrates were further explored. We found that seventy percent of SLC family genes (279/397) were differentially expressed between LUAC tissues and adjacent normal. Twenty-six genes with *p*-values < 0.05 in univariate Cox regression analysis and Kaplan-Meier survival analysis were regarded as prognosis-related SLC family genes, six of which were used to construct a prognostic signature for patient classification into high- and low-risk groups. Kaplan-Meier survival analysis in all internal and external cohorts revealed a better overall survival for patients in the low-risk group than those in the high-risk group. Univariate and multivariate Cox regression analyses indicated that the derived risk score was an independent prognostic factor for LUAD patients. Moreover, a nomogram based on the six-gene signature and clinicopathological factors was developed for clinical application. High-risk patients had lower stromal, immune, and ESTIMATE scores and higher tumor purities than those in the low-risk group. The proportions of infiltrating naive CD4 T cells, activated memory CD4 T cells, M0 macrophages, resting dendritic cells, resting mast cells, activated mast cells, and eosinophils were significantly different between the high- and low-risk prognostic groups. In all, the six-gene SLC family signature is of satisfactory accuracy and generalizability for predicting overall survival in patients with LUAD. Furthermore, this prognostics signature is related to tumor immune status and distinct immune cell infiltrates in the tumor microenvironment.

## Introduction

Lung cancer is the leading cause of cancer-related mortality worldwide (Hirsch et al., 2017). In China, lung cancer is the most common malignancy, incurring a huge economic and healthcare burden (Su and Wu, 2017; Cao and Chen, 2019). There are two main histological subtypes of lung cancer: non-small cell lung cancer (NSCLC) and small cell lung cancer (SCLC) (de Sousa and Carvalho, 2018), the former accounting for ∼85% of cases (Testa et al., 2018). NSCLC can be further classified into three subtypes: lung adenocarcinoma (LUAD), large cell carcinoma, and squamous cell carcinoma, of which LUAD is the most common, accounting for 40% of cases (Rotow and Bivona, 2017; Herbst et al., 2018). While there have been significant advances in the diagnosis and management of LUAD, clinical outcomes remain poor and 5-year overall survival (OS) is only about 15% (Wang et al., 2021).

LUAD shows high molecular heterogeneity and has a tendency to metastasize early (Devarakonda et al., 2015). It remains difficult to accurately predict outcomes for patients with LUAD using current approaches (Calvayrac et al., 2017). There is therefore an urgent need to develop more effective and robust prognostic biomarkers so that optimal and personalized therapeutic and management schemes can be developed and applied to distinct subsets of LUAD patients.

Solute carrier (SLC) proteins, the second largest family of membrane proteins in humans after G-protein-coupled receptors, have received relatively little attention over the last few years (César-Razquin et al., 2015; Pizzagalli et al., 2021). SLCs are integral cell membrane proteins localized at the cell surface and in organelle membranes (Panda et al., 2020). Functionally, SLCs transport a diverse array of substrates and participate in many essential physiological processes including nutrient uptake, ion transport, waste removal, and drug absorption and disposition (Nyquist et al., 2017; Schumann et al., 2020). SLCs are often dysregulated in human diseases, especially cancer, suggesting potential as therapeutic targets (Cormerais et al., 2018; Zhang et al., 2019; Panda et al., 2020). Indeed, many therapeutic approaches that target SLC family members such as SLC3A2 have been examined in cancer clinical trials (Lin et al., 2015). In LUAD, aberrant expression of SLC family genes has been reported to be associated with cellular proliferation and survival, and they may be useful diagnostic and prognostic biomarkers (Ikeda et al., 2015; Guo et al., 2020; Hu et al., 2020; Zhou et al., 2021). Nevertheless, the expression profiles and clinical value of SLC family members in LUAD remain largely unexplored.

Public databases including The *Cancer* Genome Atlas (TCGA) and Gene Expression Omnibus are now widely used for the discovery of prognostic biomarkers and potential therapeutic targets in many cancers, including LUAD. Here we performed an integrated analysis of SLC family genes in LUAD by: 1) identifying prognosis-related SLC genes; 2) using them to construct a prognostic signature in LUAD; 3) evaluating the prognostic accuracy of the signature in patients with LUAD; and 4) examining associations between the signature and the tumor immune microenvironment and immune cell infiltration.

## Materials and Methods

### Data Collection

Transcriptomic profiles and relevant clinical information of patients with LUAD were downloaded from the TCGA (https://portal.gdc.cancer.gov/) and GEO databases. A total of 1,036 samples were retrieved, including 535 LUAD tissues and 59 adjacent non-tumor areas from the TCGA database and 442 LUAD cases from the GSE72094 dataset. Samples without follow-up time or survival status were excluded.

### Identification of Prognosis-Related SLC Family Genes

SLC family genes were identified according to a previous study (César-Razquin et al., 2015) and the Human Gene database (https://www.genecards.org/). SLC family genes that were differentially expressed between LUAD tissues and adjacent normal tissues were screened out according to the criteria of a *p*-value < 0.05 using the *limma* package in R. Then, differentially expressed SLC genes were subjected to univariate Cox regression and Kaplan-Meier (K-M) survival analyses. Genes with *p*-values < 0.05 in both analyses were regarded as prognosis-related SLC family genes.

### Construction and Validation of the SLC Family Gene Based-Signature

To construct an SLC family gene signature, we first divided the TCGA LUAD cohort (entire cohort) into a training cohort and a testing cohort at a ratio of approximately 1:1. In the training cohort, the candidate genes were subjected to least absolute shrinkage and selection operator (lasso) regression analysis to avoid overfitting using the *glmnet* package in R. Then, stepwise multivariate Cox regression analysis was performed to determine signature genes and calculate corresponding regression coefficients. The risk score was calculated for LUAD patients according to a linear combination of the expression levels of signature genes and corresponding regression coefficients. The formula was as follows: risk score = $\overset{n}{\underset{i}{\sum }}{\text{Exp}}_{i}{\text{Coe}}_{i}$ , where Exp = expression of SLCs and Coe = the regression coefficient. The risk scores were then calculated for patients in the training, testing, entire, and external GSE72094 cohorts which, when stratified according to the median risk score in the training cohort, categorized patients in all internal and external cohorts as either high or low risk. K-M survival analysis was performed using the *survival* and *survminer* packages in R to compare OS between high- and low-risk groups. Time-dependent receiver operating characteristic curves (ROC) were implemented using the *survivalROC* package in R to evaluate the sensitivity and specificity of the SLC family signature in predicting OS in LUAD patients.

### Development of a Prognostic Nomogram in LUAD

Clinicopathological factors including gender, age, stage, and risk score based on the SLC family gene signature were integrated into a prognostic nomogram. Calibration plots were utilized to assess the predictive power of the nomogram in the TCGA LUAD cohort and external GSE72094 cohort.

### Estimating Tumor Immune Microenvironment and Immune Cell Infiltration

The stromal score, immune score, ESTIMATE score, and tumor purity in each sample were calculated using the *estimate* package in R to quantify the tumor immune microenvironment. K-M survival analysis was performed to compare the OS of patients with different stromal scores, immune scores, ESTIMATE scores, and tumor purities. The abundance of 22 immune cell subtypes was converted from the normalized gene expression matrix using the CIBERSORT algorithm. The immune cell abundance matrix was filtered with the criteria of a *p*-value < 0.05. The proportion of infiltrating immune cells in the high- and low-risk groups in the TCGA LUAD cohort and external GSE72094 cohort were compared.

### Statistical Analysis

All statistical analyses were performed in R (version R.4.1.0). A two-sided *p*-value < 0.05 was regarded as statistically significant.

## Results

### Identification of Prognosis-Related SLC Family Genes in LUAD

**FIGURE 1:**
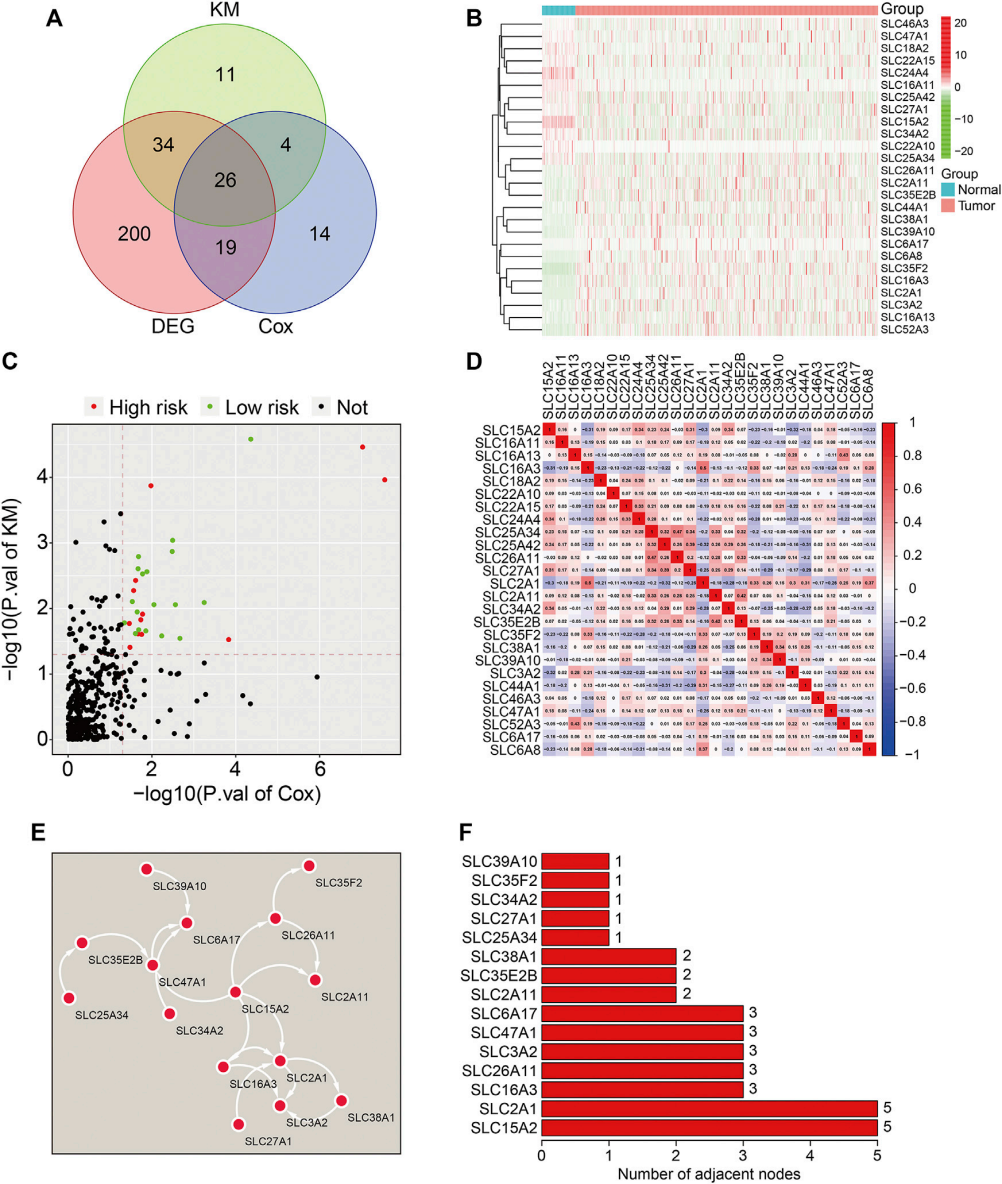
Identification of prognosis-related SLC family genes in lung adenocarcinoma. **(A)** Venn plot showing the 26 prognosis-related SLCs differentially expressed between LUAD tissues and adjacent non-tumor tissues. **(B)** The expression profiles of the 26 prognosis-related SLCs. **(C)** Volcano plot showing the prognosis-related SLCs. **(D)** Correlation heatmap of the 26 prognosis-related SLCs. **(E)** Protein-protein interaction (PPI) network for the 26 prognosis-related SLCs. **(F)** Hub genes identified in the PPI network.

Three-hundred and ninety-seven well-defined SLC family genes were identified, and their expression was extracted from the TCGA LUAD dataset. As shown in Figure 1A, 70% of SLC family genes (279/397) were differentially expressed between lung adenocarcinoma and adjacent normal tissues. Univariate Cox regression analysis and K-M survival analysis were performed to identify prognosis-related genes in the 279 differentially expressed SLC family genes; 26 genes with *p*-values < 0.05 in both analyses were regarded as prognosis-related SLC family genes (Figure 1A), and their expression is shown in Figure 1B. Of the 26 genes, 11 were risk factors with hazard ratios (HRs) > 1 and the other 15 were protective factors with HRs <1 (Figure 1C). The correlations between the 26 SLC genes based on expression are shown in Figure 1D. By constructing a protein-protein interaction network using the STRING online tool (Figure 1E), hub gene analysis revealed that *SLC15A2* and *SLC2A1* were the top two ranked genes, since they harbored the highest number of adjacent nodes (Figure 1F).

### Construction of an SLC Family-Based Signature in the TCGA LUAD Training Cohort

**FIGURE 2:**
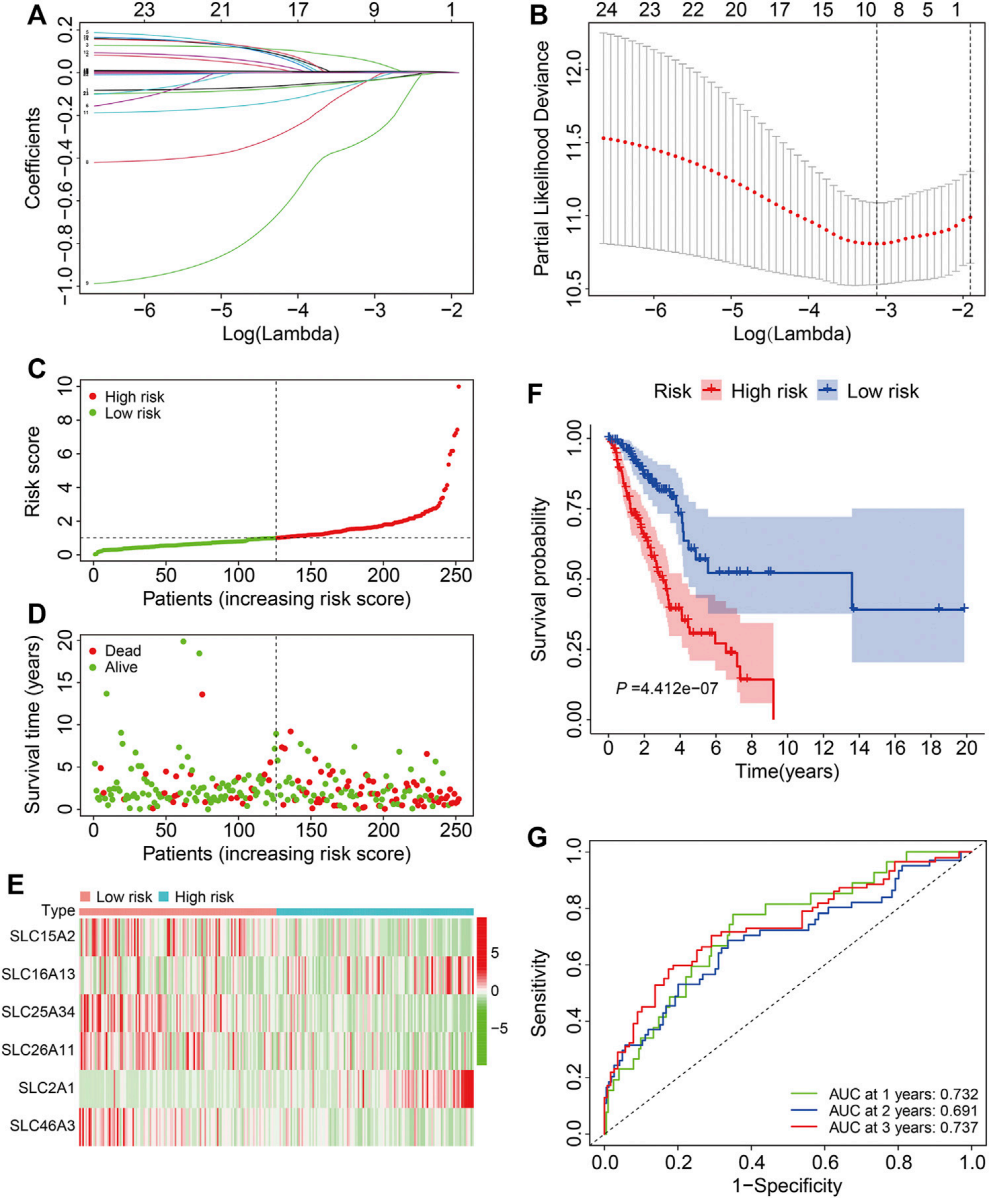
Construction of a six-gene SLC family signature in the training cohort. **(A)** The association between coefficients of genes and log (lamba). **(B)** The association between deviance and log (lamba). **(C)** The risk score distribution in the training cohort. **(D)** The vital status and follow-up time of patients in the high- and low-risk groups. **(E)** The expression profiles of the six SLC genes. **(F)** Kaplan-Meier survival analysis of the high- and low-risk groups. **(G)** Time-dependent ROC curve analysis of the six-gene signature for predicting overall survival in the training cohort.

The 26 prognosis-related SLC genes were subjected to lasso regression analysis in training cohort to avoid overfitting (Figure 2A). Then, stepwise multivariate Cox regression analysis was performed to establish a risk signature based on six SLC genes (*SLC15A2*, *SLC16A13*, *SLC25A34*, *SLC26A11*, *SLC2A1*, and *SLC46A3*) (Figure 2B). The risk score was calculated according to a linear combination of the expression levels of the six SLC genes and corresponding regression coefficients (Table 1), where the risk score = *SLC15A2* × (−0.0904) + *SLC16A13* × 0.1379 + *SLC25A34* × (−0.6020) + *SLC26A11* × (−0.1386) + *SLC2A1* × 0.0063 + *SLC46A3* × (−0.0891).

**TABLE 1:** Details of the six SLC genes in the prognostic model.

Gene name	Coefficient	HR	HR.95 L	HR.95H	*P*
*SLC15A2*	−0.0904	0.9135	0.8392	0.9944	0.0367
*SLC16A13*	0.1379	1.1478	1.0277	1.2819	0.0145
*SLC25A34*	−0.6020	0.5477	0.2466	1.2167	0.1393
*SLC26A11*	−0.1386	0.8706	0.7513	1.0088	0.0652
*SLC2A1*	0.0063	1.0063	1.0012	1.0115	0.0165
*SLC46A3*	−0.0891	0.9147	0.8532	0.9808	0.0122

According to median value of the risk score, patients in the training cohort were separated into high- and low-risk groups (Figure 2C) and, as expected, patients with higher risk scores were more likely to have died and lived for a shorter period (Figure 2D). The expression profiles of the six SLC genes in high- and low-risk groups are shown in Figure 2E. K-M analysis demonstrated that patients in the low-risk group lived longer than those in the high-risk group (Figure 2F). The 1-, 3-, and 5-years OS AUC values were 0.732, 0.691, and 0.737, respectively (Figure 2G), suggesting that the SLC family-based signature accurately predict survival outcomes in LUAD patients.

### Validation of the Six-Gene SLC Family Signature in Internal Cohorts

**FIGURE 3:**
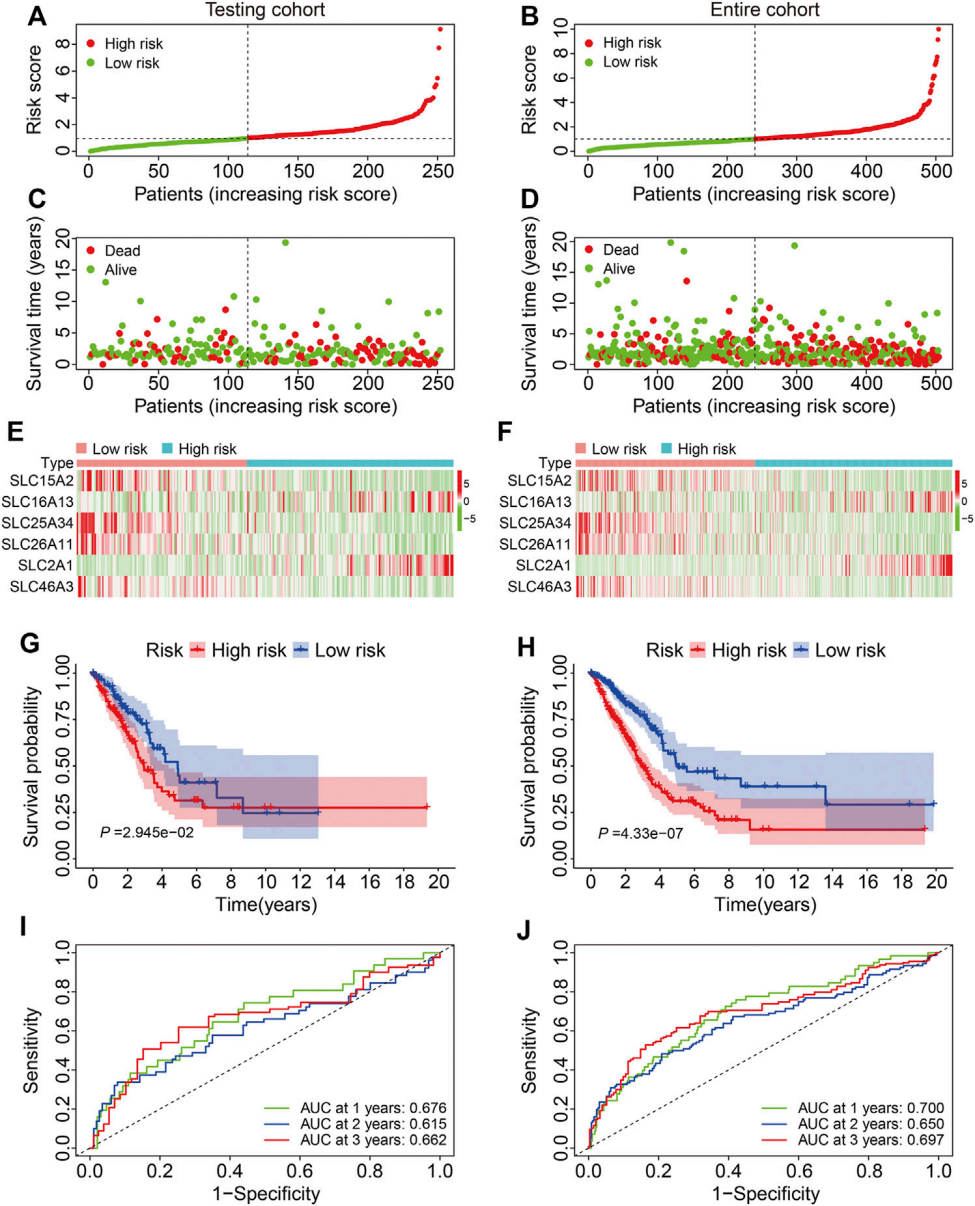
Validation of the six-gene SLC family signature in internal cohorts. **(A,B)** The risk score distribution in the testing cohort and entire cohort. **(C,D)** The vital status and follow-up time of patients in the testing cohort and entire cohort. **(E,F)** The expression profiles of the six SLC genes in the testing cohort and entire cohort. **(G,H)** Kaplan-Meier survival analysis of high- and low-risk groups in the testing cohort and entire cohort. **(I,J)** Time-dependent ROC curve analysis of the six-gene signature for predicting overall survival in the testing cohort and entire cohort.

We first validated the SLC family-based signature in internal cohorts including a testing cohort and the entire cohort. Risk scores were calculated for patients in the test and entire cohort as above and stratified into high- and low-risk groups according to the median risk score in training cohort (Figures 3A,B). The vital status and follow-up time of patients in the test and entire cohorts are shown in Figures 3C,D, and the expression patterns of the six SLC genes in patients with different risk scores are shown in Figures 3E,F. K-M survival analysis confirmed worse OS in the high-risk group than the low-risk group in both cohorts (Figures 3G,H). The AUC values of the ROC curves were 0.676 at 1 year, 0.615 at 3 years, and 0.662 at 5 years in the test cohort (Figure 3I) and 0.700 at 1 year, 0.650 at 3 years, and 0.697 at 5 years in the entire cohort (Figure 3J).

### Validation of Six Gene SLC Family Signature in the External GSE72094 Cohort

**FIGURE 4:**
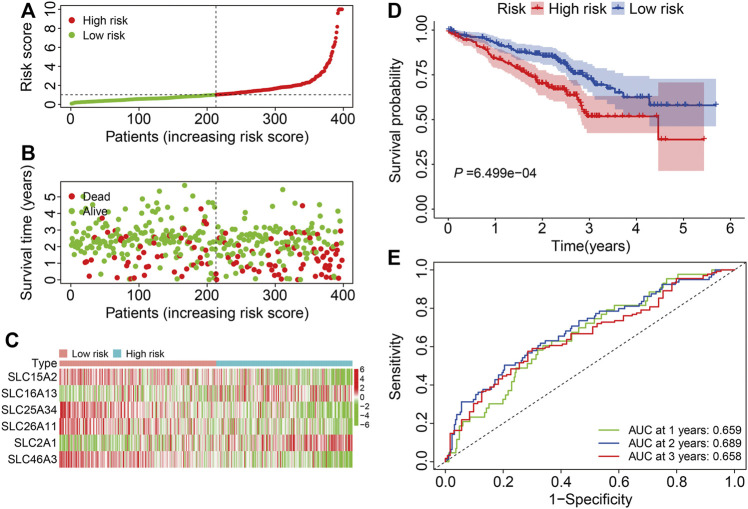
Validation of the six-gene SLC family signature in the external GSE72094 cohort. **(A)** The risk score distribution in the GSE72094 cohort. **(B)** The vital status and follow-up time of patients in the GSE72094 cohort. **(C)** The expression profiles of the six SLC genes in the GSE72094 cohort. **(D)** Kaplan-Meier survival analysis of high- and low-risk groups in the GSE72094 cohort. **(E)** Time-dependent ROC curve analysis of the six-gene signature for predicting overall survival in the GSE72094 cohort.

We next verified the accuracy and generalizability of the derived signature in the external GSE72094 dataset by defining patients as high or low risk as above (Figure 4A). The vital status and follow-up time of patients in the external cohort are shown in Figure 4B, and the expression profiles of the six SLC genes are shown in Figure 4C. Again, K-M analysis revealed large and significant differences in OS between high- and low-risk groups (Figure 4D). The 1-, 3-, and 5-years OS AUC values were 0.695, 0.689, and 0.658, respectively (Figure 4E).

### Stratified Analysis of the Six-Gene Signature Based on Clinicopathological Features

**FIGURE 5:**
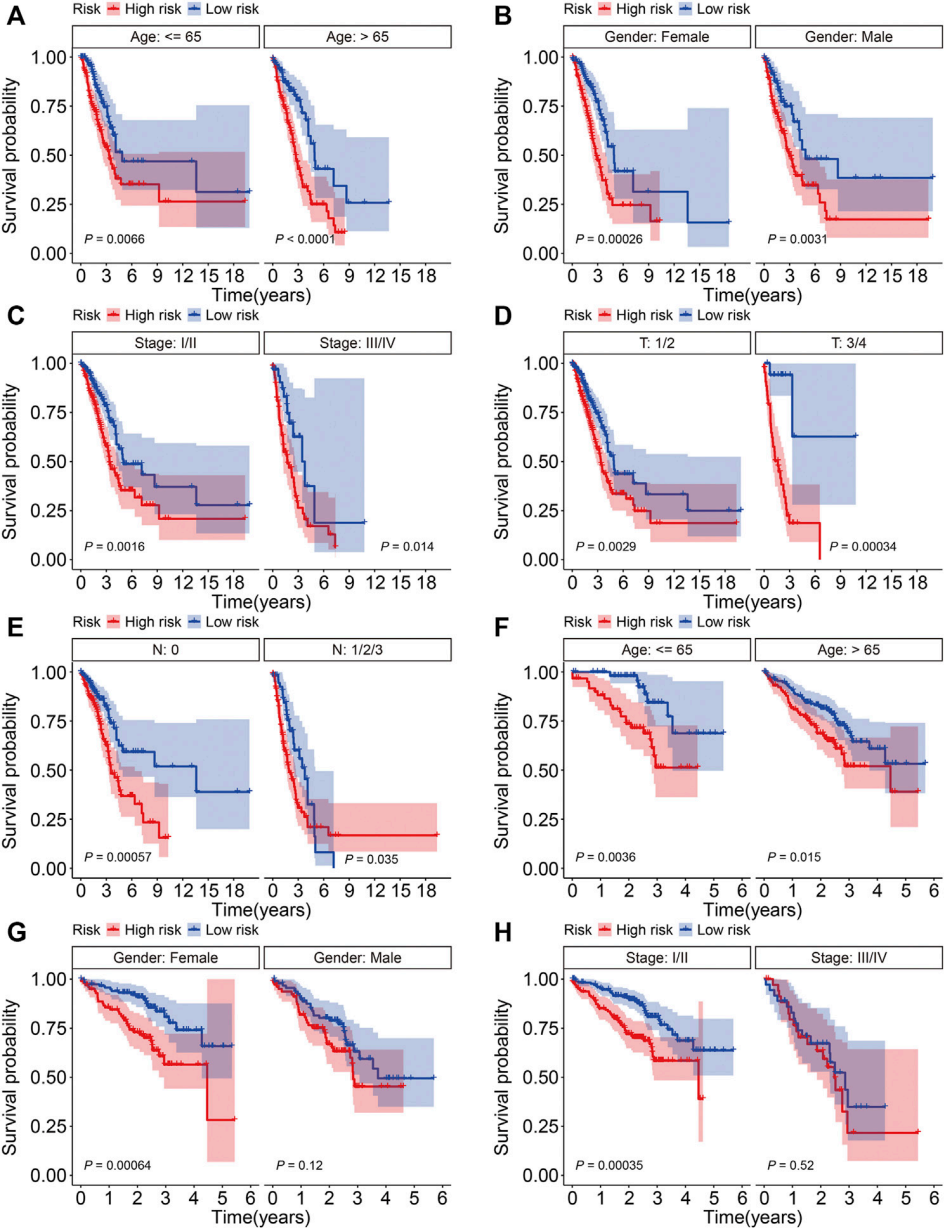
Kaplan-Meier survival analysis in subgroups classified by age **(A)**, gender **(B)**, stage **(C)**, and T **(D)** and N status **(E)** in the TCGA cohort. Kaplan-Meier survival analysis in subgroups classified by age **(F)**, gender **(G)**, and stage **(H)** in the GSE72094 cohort.

To further assess the prognostic power of the six-gene signature, patients in the TCGA and GSE72094 cohorts were classified into various subgroups based on clinicopathological features including age (≤65, >65), gender (female, male), stage (stage I/II, stage III/IV), T (tumor) status (T 1/2, T 3/4), and N (node) status (N 0, N 1/2/3). The OS of patients with different risk scores was compared in subgroups stratified by age, gender, stage, and T and N status in the TCGA cohort, which showed that patients in the low-risk group survived longer in all subgroups (Figures 5A–E). In a similar analysis of the GSE72094 cohort stratified according to age, gender, and stage, while the OS of patients was worse in the high-risk group than the low-risk group in these subgroups, the differences were not significantly different in terms of being male and patients with stage III/IV tumors (Figures 5F–H).

### Independent Prognostic Analysis of the Six-Gene Signature and Development of a Nomogram

**FIGURE 6:**
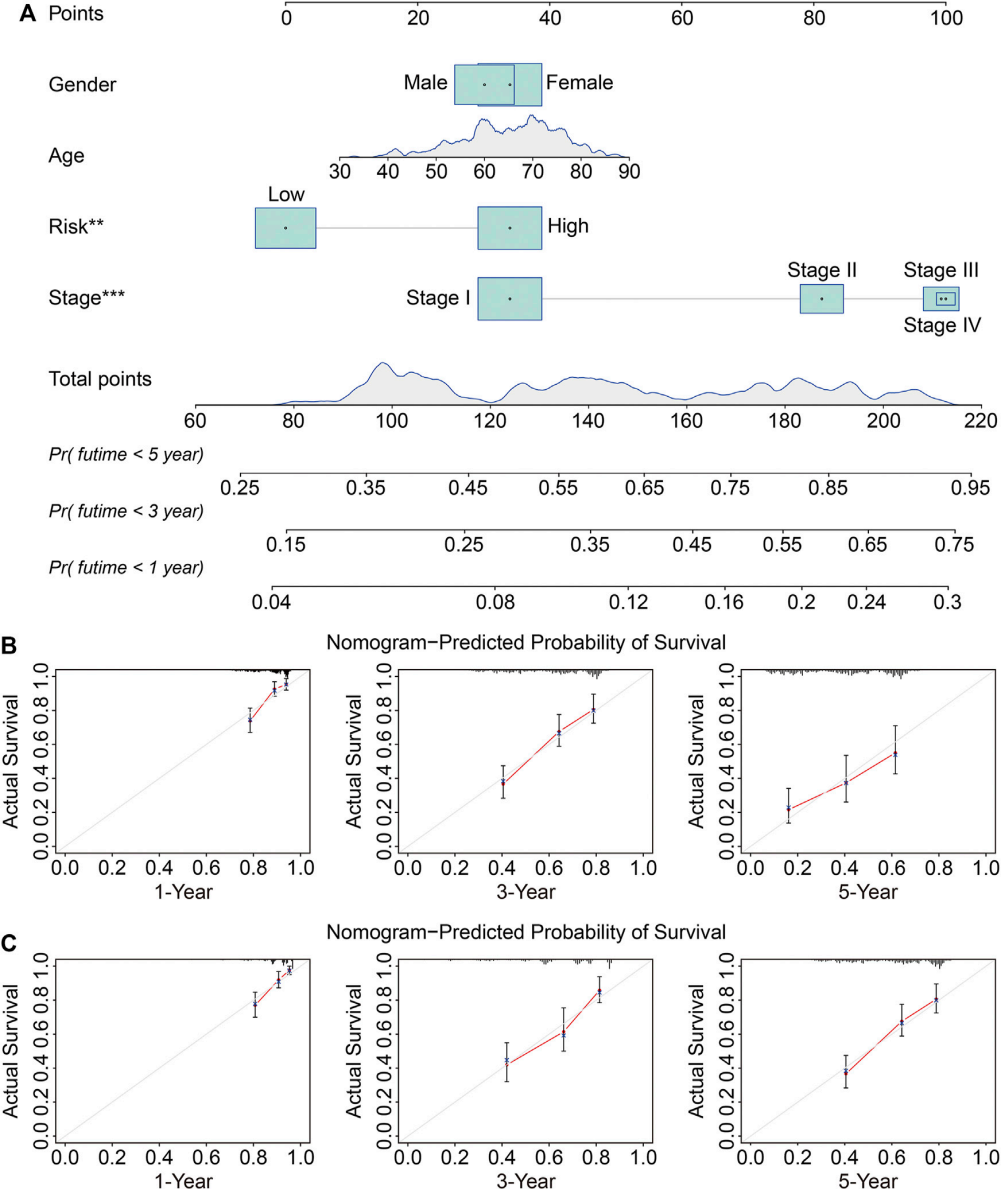
Construction and validation of a nomogram in the TCGA and GSE72094 cohorts. **(A)** The nomogram based on the six-gene signature and clinicopathological factors including gender, age, and stage. **(B,C)** Calibration curves for the nomogram predicting 1-, 3-, and 5-year survival in LUAD patients from the TCGA **(B)** and GSE72094 **(C)** cohorts.

We next conducted univariate and multivariate Cox regression analyses to evaluate the influence of clinicopathological factors and SLC risk score on the OS of LUAD patients in the TCGA and GSE72094 cohorts. As shown in Table.2 and Table.3, the SLC risk score was the only independent prognostic factor in both cohorts. Moreover, we established a prognostic nomogram in the TCGA cohort that included the risk score and clinicopathological factors including gender, age, and stage (Figure 6A). The 1-, 3-, and 5-years OS calibration curves in the TCGA and GSE72094 cohorts were close to the ideal curve, suggesting that the nomogram could accurately predict outcomes of LUAC patients (Figures 6B,C).

**TABLE 2:** Univariable and multivariable analysis of the six-gene signature and clinical factors in the TCGA cohort.

Variables	Univariable analysis				Multivariable analysis			
	HR	95% CI of HR		*p*	HR	95% CI of HR		*p*
		Lower	Upper			Lower	Upper	
Gender (Female vs. Male)	1.1116	0.8252	1.4974	0.4863	0.9096	0.6676	1.2394	0.5483
Age (≤65 vs. > 65)	1.2053	0.8933	1.6261	0.2218	1.3105	0.9642	1.7812	0.0842
Stage (I/II vs. III/IV)	2.4050	1.7518	3.3018	0.0000	1.1927	0.7833	1.8160	0.4114
T (T 1/2 vs. T 3/4)	2.2803	1.5619	3.3291	0.0000	1.5861	1.0371	2.4257	0.0333
N (N 0 vs. N 1/2/3)	2.4961	1.8508	3.3664	0.0000	2.0760	1.4424	2.9880	0.0001
Risk (High vs. Low)	1.3046	1.2144	1.4014	0.0000	1.2657	1.1665	1.3733	0.0000

**TABLE 3:** Univariable and multivariable analysis of the six-gene signature and clinical factors in the GSE72094 cohort.

Variables	Univariable analysis				Multivariable analysis			
	HR	95% CI of HR		*p*	HR	95% CI of HR		*p*
		Lower	Upper			Lower	Upper	
Gender (Female vs. Male)	1.5470	1.0653	2.2465	0.0219	1.7539	1.1983	2.5673	0.0038
Age (≤65 vs. > 65)	1.4043	0.9147	2.1559	0.1206	1.3465	0.8710	2.0813	0.1807
Stage (I vs. II/III/IV)	1.6249	1.3601	1.9412	0.0000	1.6628	1.3855	1.9957	0.0000
Risk (High vs. Low)	1.0901	1.0409	1.1417	0.0003	1.0951	1.0447	1.1479	0.0002

### Association of the SLC Family-Based Signature With the Tumor Immune Microenvironment and Immune Cell Infiltration

**FIGURE 7:**
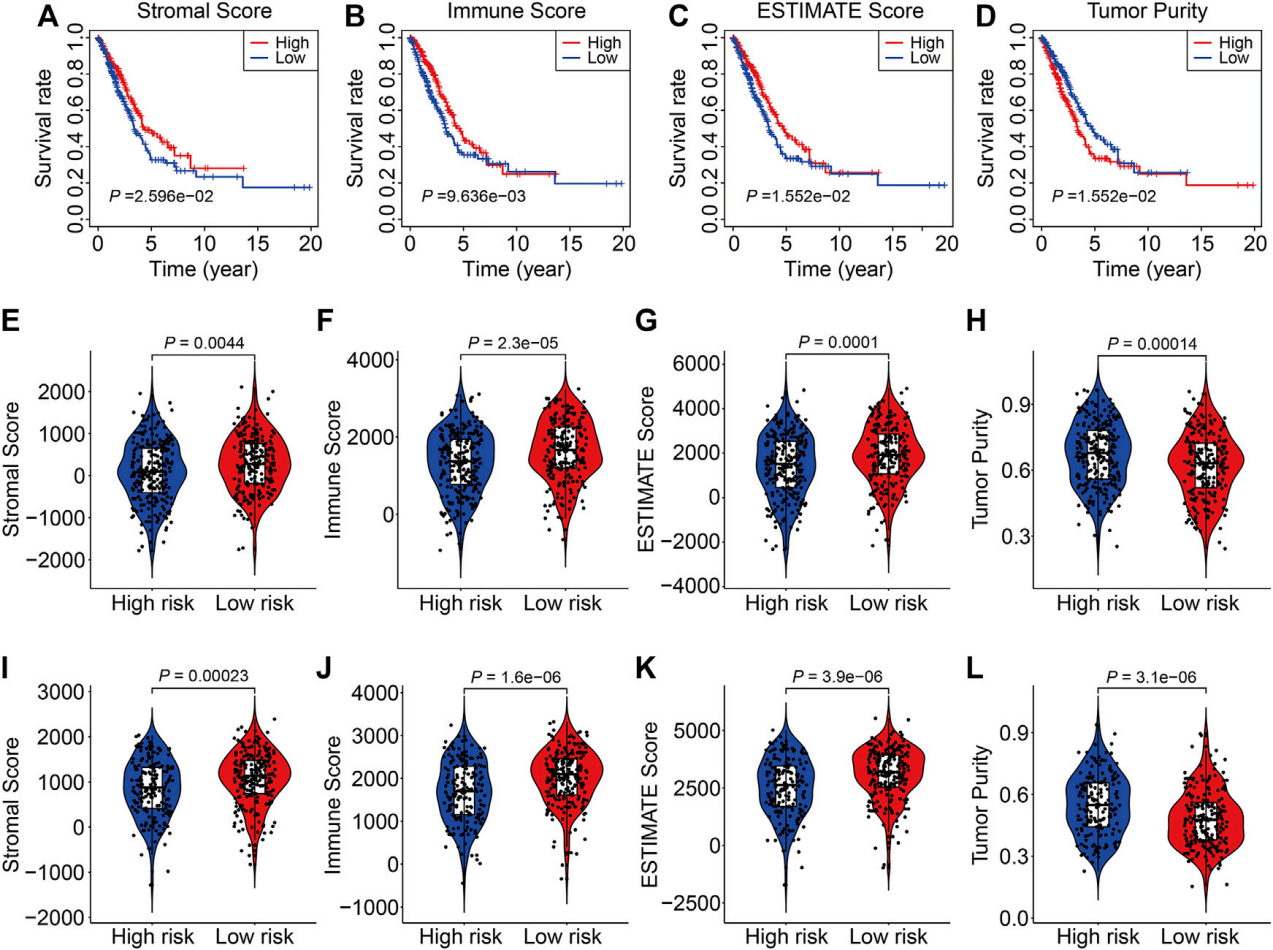
The six-gene SLC family signature was correlated with the tumor immune microenvironment. **(A–D)** Kaplan-Meier survival analysis of the stromal score, immune score, ESTIMATE scores, and tumor purity in the TCGA cohort. **(E–H)** Comparison of stromal score, immune score, ESTIMATE scores, and tumor purity between high- and low-risk groups in the TCGA cohort. **(I–L)** Comparison of stromal score, immune score, ESTIMATE scores, and tumor purity between high- and low-risk groups in the GSE72094 cohort.

To explore associations between the SLC family-based signature and the immune microenvironment, we employed the ESTIMATE algorithm to quantify the stromal score, immune score, ESTIMATE score, and tumor purity in each TCGA and GSE72094 sample. K-M survival analysis revealed that cases with lower stromal, immune, or ESTIMATE scores had a worse OS than those with higher scores (Figures 7A–C). Conversely, patients with lower tumor purity had a better prognosis than those with higher tumor purity (Figure 7D). These analyses suggest that the tumor immune microenvironment is closely associated with outcomes in LUAC patients. Consistently, patients in the high-risk group in both the TCGA and GSE72094 cohorts had lower stromal, immune, or ESTIMATE scores (Figures 7E–G,I–K) and higher tumor purity (Figure 7H–L) than those in the low-risk group.

**FIGURE 8:**
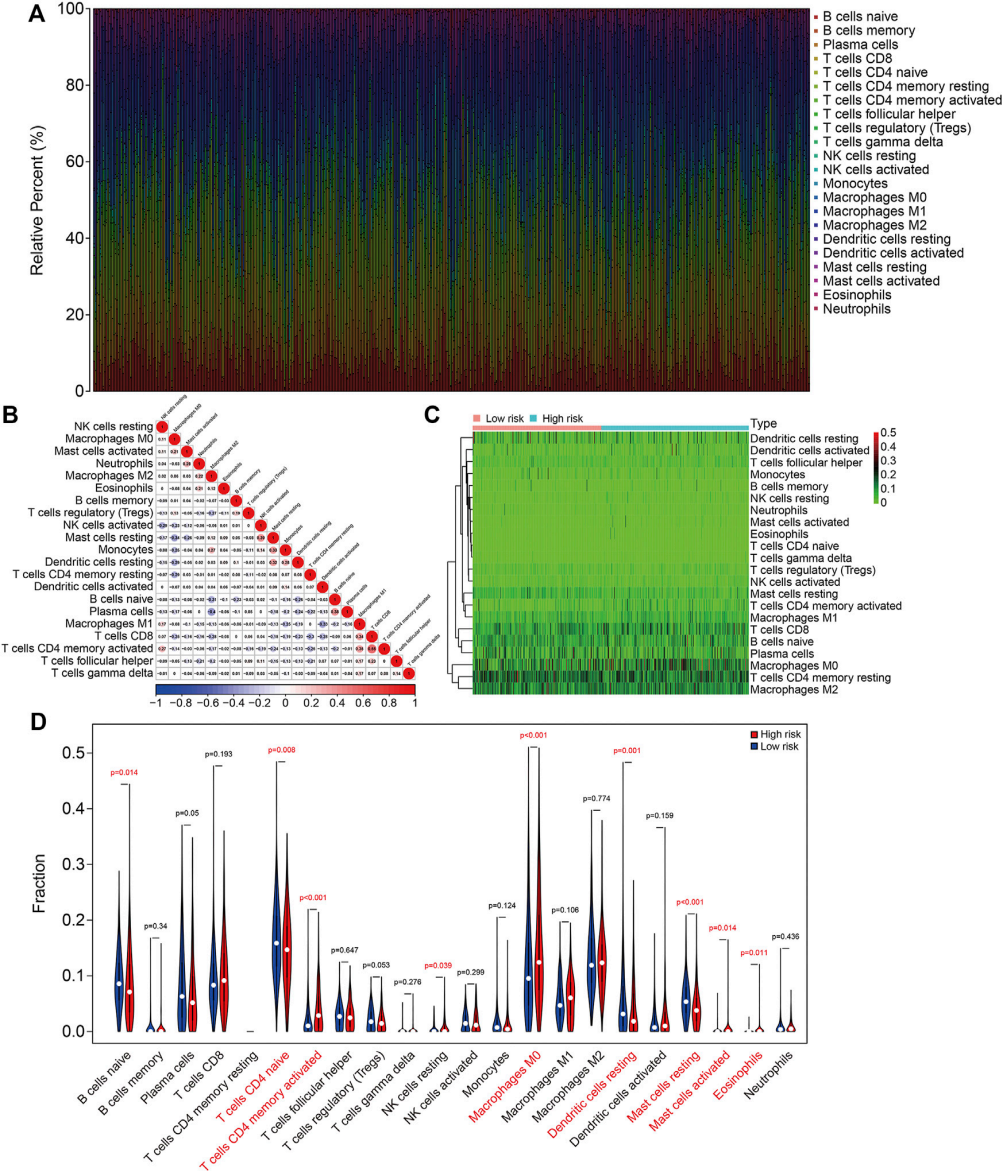
The six-gene SLC family signature was correlated with tumor immune cell infiltration in the TCGA lung adenocarcinoma cohort. **(A)** Abundance of 22 immune cell types in the TCGA cohort. **(B)** The correlations between the infiltrating immune cells in the TCGA cohort. **(C,D)** Heatmap and violin plot showing the comparison of infiltrating immune cells in the high- and low-risk groups in the TCGA cohort.

To further characterize the immune microenvironment in LUAC, the proportions of 22 types of infiltrating immune cell were quantified using the ESTIMATE algorithm (TCGA cohort, Figure 8A and GSE72094 cohort, Supplementary Figure S1A). The correlations between the infiltrating immune cells in the TCGA cohort are shown in Figure 8B and for the GSE72094 cohort in Supplementary Figure S1B. The proportions of infiltrating naive CD4 T cells, activated memory CD4 T cells, M0 macrophages, resting dendritic cells, resting mast cells, activated mast cells, and eosinophils were significantly different between the high- and low-risk groups for both cohorts (Figures 8C,D, Supplementary Figures S1C,D).

### Expression and Survival Analysis of the Six SLC Family Genes

**FIGURE 9:**
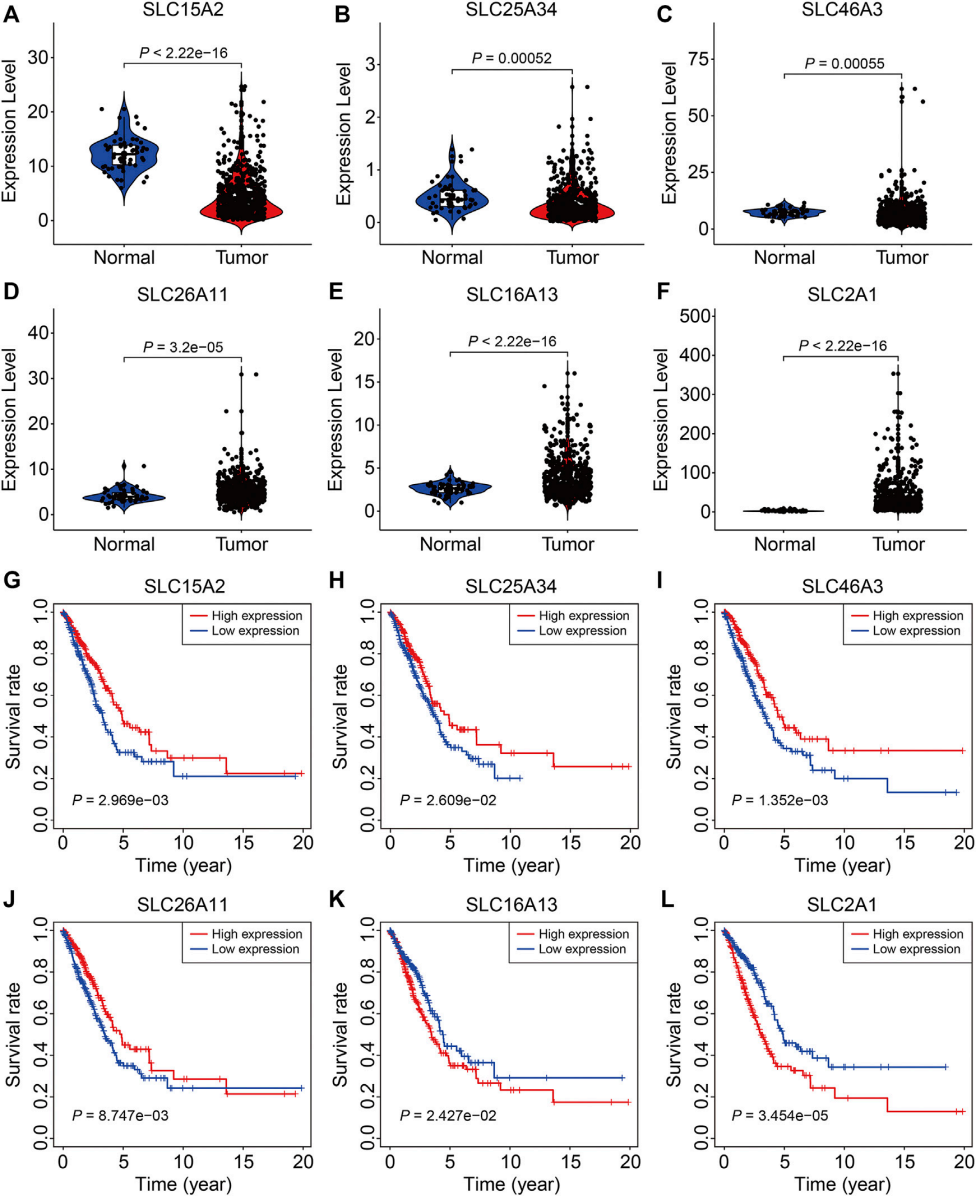
The expression and survival analyses of the six SLC family genes. **(A–F)** The expression of *SLC15A2*, *SLC25A34*, *SLC46A3*, *SLC26A11*, *SLC16A13*, and *SLC2A1* in LUAD tissues and adjacent non-tumor tissues. **(G–L)** Kaplan-Meier survival analysis of *SLC15A2*, *SLC25A34*, *SLC46A3*, *SLC26A11*, *SLC16A13*, and *SLC2A1* in the TCGA cohort.

Finally, we analyzed the expression and conducted Kaplan-Meier survival analysis of the six SLC family genes in the TCGA and GSE72094 datasets. As shown in Figures 9A–F, compared to adjacent normal tissues, the expression of *SLC15A2*, *SLC25A34*, and *SLC46A3* were significantly lower and the expression of *SLC26A11*, *SLC16A13*, and *SLC2A1* significantly higher in LUAC tissues, which were consistent with the results analyzed in the UALCAN (http://ualcan.path.uab.edu/) online tool (Pan et al., 2021). K-M survival analysis indicated that lower expression of *SLC15A2*, *SLC25A34*, *SLC46A3*, and *SLC26A11* and higher expression of *SLC16A13* and *SLC2A1* were associated with a worse prognosis in the TCGA cohort (Figures 9G–L). In the GSE72094 cohort, lower expression of *SLC15A2* and *SLC26A11* and higher *SLC2A1* expression were associated with worse OS (Supplementary Figures S2A,D,F), while the expression of *SLC25A34*, *SLC46A3*, and *SLC16A13* was not associated with prognosis (Supplementary Figures S2B,C,E).

## Discussion

With the development of high-throughput sequencing technology and its popularization in cancer research, an increasing number of prognostic biomarkers and therapeutic targets have been identified in various malignancies through bioinformatics analyses (Reuter et al., 2015; Hong et al., 2020). Re-analyzing public datasets is now recognized as a valuable way to identify diagnostic or prognostic biomarkers in caner (Sun et al., 2020; Deng et al., 2021). In a previous study, Zhang et al. (2020) conducted a comprehensive molecular analysis of TNF family genes in LUAD using TCGA and GEO datasets and developed a five-gene TNF family signature that predicted OS in LUAD patients. Jiang et al. (2021) constructed a prognostic signature in LUAD based on cell cycle-related genes and further verified the accuracy of the signature in two independent GEO datasets. This approach provides an avenue for the identification of prognostic genes in LUAD.

Although SLC proteins have been relatively understudied over the past few years, they are now known to play vital roles in essential biological processes and human diseases (César-Razquin et al., 2015; Rives et al., 2017; Schaller and Lauschke, 2019). In cancer, dysregulation of SLC proteins has been shown to be oncogenic (Xie et al., 2011; Mohelnikova-Duchonova et al., 2013; Bhutia et al., 2016; Sutherland et al., 2020). Many SLCs have been reported to be aberrantly expressed in LUAD and might serve as prognostic biomarkers (Ikeda et al., 2015; Lehrer and Rheinstein, 2018). For example, elevated expression of SLC2A1 was associated with a poorer prognosis in LUAD patients (Guo et al., 2020), while increased SLC18A1 expression was associated with significantly increased survival in LUAD patients (Lehrer and Rheinstein, 2018). Additionally, aberrant SLC expression contributes to accelerated cell proliferation and invasion through diverse mechanisms. The amino acid transporter SLC38A3 was upregulated in metastatic NSCLC cells and was associated with prognosis in NSCLC patients. Functional experiments suggested that SLC38A3 overexpression promotes epithelial-mesenchymal transition and accelerates tumor metastasis by regulating glutamine and histidine transport (Wang et al., 2017). SLC39A5, a membrane transporter responsible for the dynamic balance of zinc, was upregulated in LUAD tissues compared with adjacent non-tumor lung tissues, and higher expression of SLC39A5 predicted poor survival in LUAD patients. Functionally, SLC39A5 promoted cell proliferation by accelerating cell cycle transition and inhibiting apoptosis, which was mediated by the activation of PI3K/AKT signaling (Liu et al., 2021). Taken together, these studies indicate that SLC proteins are associated with the occurrence and progression of LUAD.

Given the important role of SLCs in lung adenocarcinoma, we performed an integrated analysis of SLC family genes in LUAD. To our surprise, over 70% of SLC family genes were differentially expressed in LUAD tissues compared to adjacent non-tumor tissues. Using univariate Cox regression analysis and K-M survival analysis to identify robust prognosis-related genes among these differentially expressed SLCs, we screened out a total of 26 SLC genes. This allowed us to develop a six-gene prognostic signature and stratify patients into high- and low-risk groups. Analysis of patients in all internal and external cohorts revealed that overall survival was consistently better in the low-risk group than in the high-risk group, suggesting satisfactory accuracy and generalizability of the SLC-based signature for prognostication of LUAD patients. Furthermore, the sensitivity and specificity of the prognostic signature were confirmed in time-dependent ROC analysis. Compared with previously reported prognostic signatures in LUAD (Yi et al., 2021; Zhang et al., 2021), we found that the specificity and accuracy of our six-gene signature was similar to these prognostic signatures. We also stratified patients into various subgroups based on clinicopathological features including age, gender, stage, and T and N status, and K-M survival analysis suggested patient survival in the low-risk group was consistently better that in the high-risk group for almost all subgroups. Thus, our novel SLC-based signature is a clinically useful prognostic biomarker for LUAD patients. Moreover, our prognostic nomogram comprising the risk score and clinicopathological factors including gender, age, and stage might be practically helpful for clinical decision-making and personalizing the management of LUAD patients. Additionally, we found that the immune microenvironment in LUAD tissues was closely associated with overall survival and that high- and low-risk groups exhibited different immune statuses and distinctive immune cell proportions. Previous studies have indicated that SLC members were regulated by TGFβ1 (Kim et al., 2020), and a bispecific antibody of TGF-β and PD-L1 showed a potent and durable antitumor activity by normalizing tumor immune microenvironment and enhanced anti-tumor immune response. Thus, we could speculate that the SLC-based signature might also be useful to predict the effect of immunotherapy and SLC members were potential downstream targets that mediated the effect of TGFβ1 on tumor immune response.

The six SLC genes included *SLC15A2*, *SLC25A34*, *SLC46A3*, *SLC16A13*, *SLC2A1*, and *SLC26A11*. Of these six genes, the expression of *SLC25A34*, and *SLC46A3* was significantly lower in LUAD tissues compared with adjacent normal and lower expression was associated with a poorer prognosis in LUAD patients, suggesting a tumor suppressive effect. Up to now, the role of SLC25A34 is still unclear. SLC46A3, localized to the lysosome, is reported to be responsible for the modulation of intracellular copper levels (Kim et al., 2021). Forced expression of SLC46A3 resulted in decreased mitochondrial membrane potential and abnormal mitochondria morphology consistent with lower copper levels. In hepatocellular carcinoma, increased expression of SLC46A3 inhibited cell proliferation, migration, and invasion (Zhao et al., 2019). Moreover, higher expression of SLC46A3 could favor a better clinical prognosis for patients with HCC, ameliorate sorafenib resistance, and improve drug response. The expression of *SLC16A13* and *SLC2A1* was higher in LUAD tissues and their expression was associated with a poorer prognosis, so these two genes might be oncogenic. SLC16A13 is a lactate transporter expressed at the plasma membrane and a potential target for the treatment of type 2 diabetes and non-alcoholic fatty liver disease (Schumann et al., 2021), while its role in tumor needs to be further explored. *SLC2A1*, also known as glucose transporter type 1 (GLUT1), is well characterized in cancer and is associated with tumor progression and metastasis (Yan et al., 2015; Nagarajan et al., 2017; Xiao et al., 2018), including in LUAD where is has previously been shown to be upregulated and associated with poorer prognosis (Guo et al., 2020). SLC26A11 is a chloride transporter that can transport chloride and increase the rate of acid extrusion (Xu et al., 2011). Here, we found that *SLC26A11* was upregulated in LUAD tissues, while higher expression of *SLC26A11* was associated with a better prognosis in the TCGA cohort. Of note, the higher expression of *SLC26A11* in LUAD tissues was inconsistent with its predictive performance in LUAD. Thus, the role of *SLC26A11* in LUAD needed to be further investigated. *SLC15A2*, a peptide transporter, is widely expressed in the lungs, kidneys, brain, and eye (Biegel et al., 2006; Kamal et al., 2008; Lu et al., 2016) and is highly expressed in human glioma cells, where it mediates carnosine uptake (Oppermann et al., 2019), although its role in cell proliferation requires further exploration. In lung cancer, *SLC15A4*, the analog of *SLC15A2*, was associated with survival and cell division regulation (Huang et al., 2021). Here, we found that *SLC15A2* expression was lower in LUAD tissues, and lower expression was associated with a poorer prognosis in LUAD patients in two independent cohorts. Therefore, the role of *SLC15A2* in LUAD occurrence and development requires further exploration.

Our study has some limitations. First, all the cases in our analysis were from public databases and were retrospective, so validation of the SLC-based signature in prospective samples is needed. Second, the functional role of the six prognostic SLC genes, especially *SLC15A2*, needs further exploration *in vitro* and *in vivo*.

In conclusion, our six-gene signature based on SLC family members is of satisfactory accuracy and generalizability for predicting survival outcomes in LUAD patients. Furthermore, the signature is related to tumor immune status and distinct immune cell infiltrates in the tumor microenvironment.

## Data Availability

The datasets presented in this study can be found in online repositories. The names of the repository/repositories and accession number(s) can be found in the article/Supplementary Material.
