# Supplementary figures and images for: Identification of a Six-Gene SLC Family Signature With Prognostic Value in Patients With Lung Adenocarcinoma
Source: Front Cell Dev Biol. 2021 Dec 15;9:803198. doi: 10.3389/fcell.2021.803198 (PMC8714960; doi:10.3389/fcell.2021.803198)

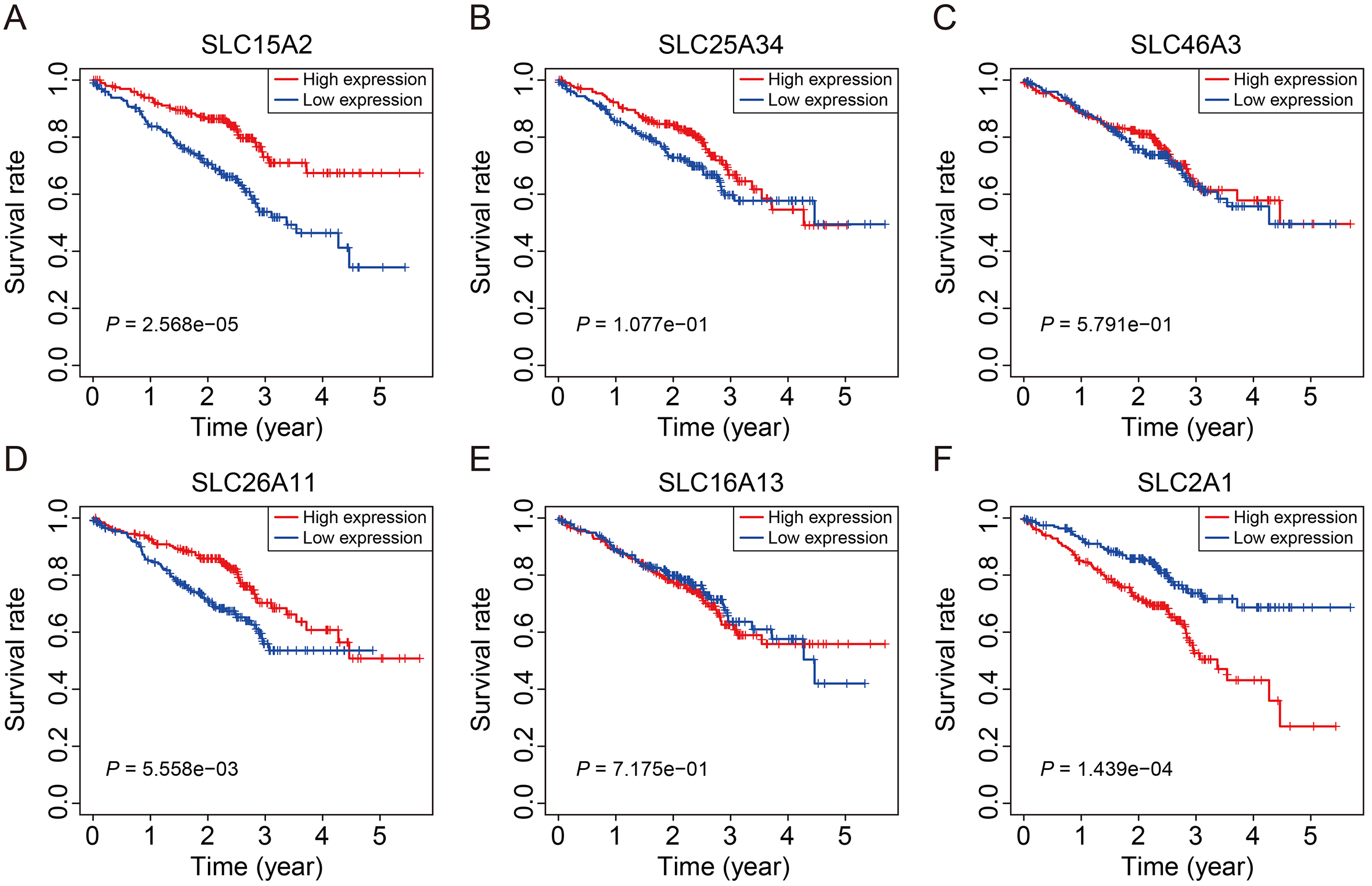

Supplement: Supplementary file 1 [file Image2.TIF]

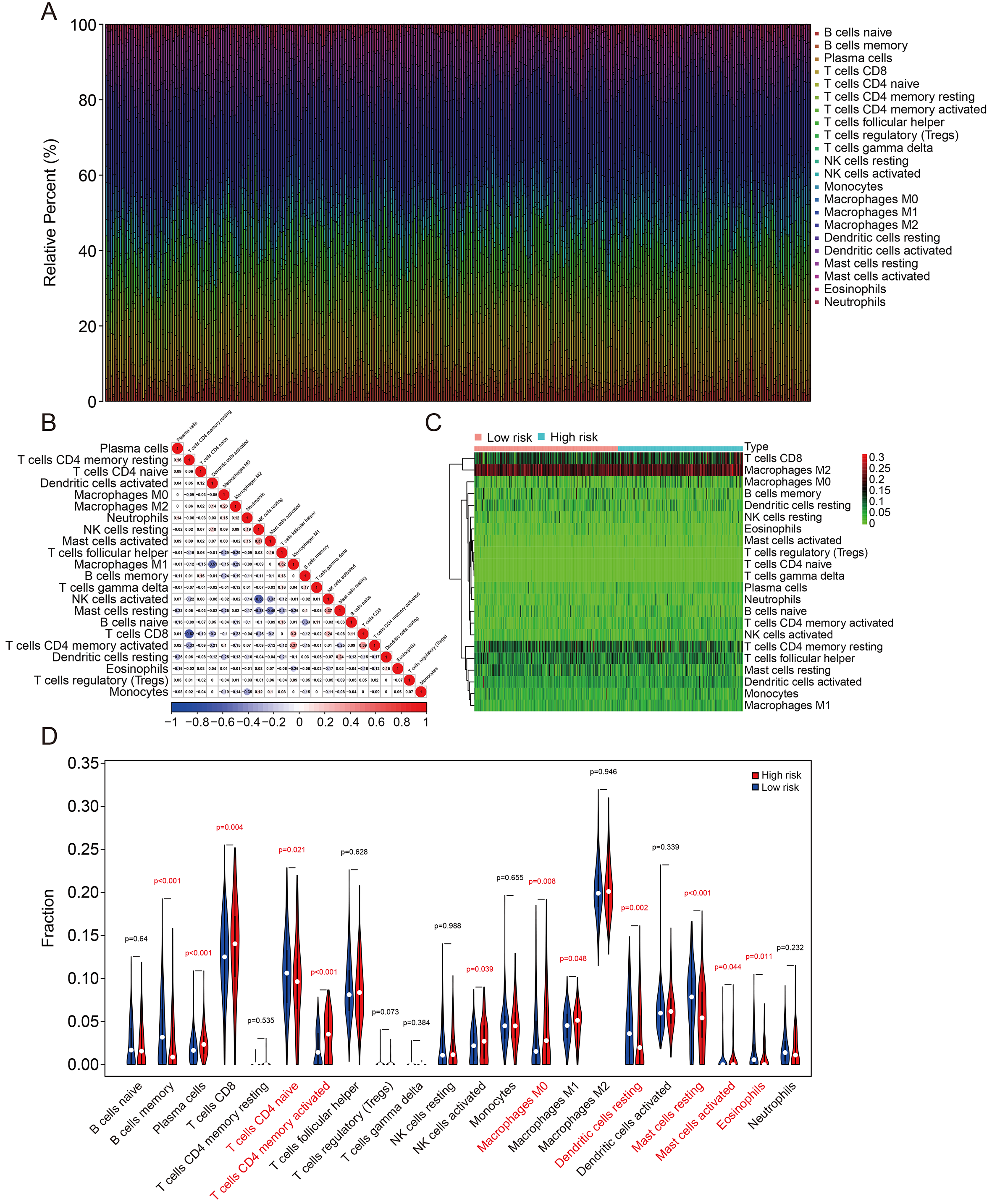

Supplement: Supplementary file 2 [file Image1.TIF]
